# The Functional Vision Protection Effect of Danshensu via Dopamine D1 Receptors: In Vivo Study

**DOI:** 10.3390/nu13030978

**Published:** 2021-03-17

**Authors:** Yun-Wen Chen, Yun-Ping Huang, Pei-Chang Wu, Wei-Yu Chiang, Ping-Hsun Wang, Bo-Yie Chen

**Affiliations:** 1 Department of Ophthalmology, Kaohsiung Chang Gung Memorial Hospital, Chang Gung University College of Medicine, Kaohsiung 88301, Taiwan; curiouscat@cgmh.org.tw (Y.-W.C.); wooopc@gmail.com (P.-C.W.); lcwarm021@gmail.com (W.-Y.C.); 2 Department of Optometry, Chung Shan Medical University, Taichung 40201, Taiwan; a0916782563@gmail.com (Y.-P.H.); epyon522121@gmail.com (P.-H.W.); 3 Department of Ophthalmology, Chung Shan Medical University Hospital, Taichung 40201, Taiwan

**Keywords:** danshensu, retina, functional vision, visual contrast sensitivity function

## Abstract

Danshensu, a traditional herb-based active component (*Salvia miltiorrhiza* Bunge), has garnered attention, due to its safety, nutritional value, and antioxidant effects, along with cardiovascular-protective and neuroprotective abilities; however, its effect on the retinal tissues and functional vision has not been fully studied. The objective of this study was to analyze the protective effect of danshensu on retinal tissues and functional vision in vivo in a mouse model of light-induced retinal degeneration. High energy light-evoked visual damage was confirmed by the loss in structural tissue integrity in the retina accompanied by a decline in visual acuity and visual contrast sensitivity function (VCSF), whereas the retina tissue exhibited severe Müller cell gliosis. Although danshensu treatment did not particularly reduce light-evoked damage to the photoreceptors, it significantly prevented Müller cell gliosis. Danshensu exerted protective effects against light-evoked deterioration on low spatial frequency-based VCSF as determined by the behavioral optomotor reflex method. Additionally, the protective effect of danshensu on VCSF can be reversed and blocked by the injection of a dopamine D1 receptor antagonist (SCH 23390). This study demonstrated that the major functional vision promotional effect of danshensu in vivo was through the dopamine D1 receptors enhancement pathway, rather than the structural protection of the retinas.

## 1. Introduction

*Salvia miltiorrhiza* Bunge (*S. miltiorrhiza* or Danshen), a traditional medicinal herb in many Asian countries, has been widely used to improve blood circulation, resolve blood stasis, attenuate atherosclerosis, treat myocardial ischemia-reperfusion injury, renal and hepatic dysfunction, and coronary heart disease [1,2]. Accumulating evidence suggests that the active components of *S. miltiorrhiza* also possess general cerebrovascular and neuropharmacological effects [2,3,4]. The chemical constituents present in *S. miltiorrhiza* are classified into water-soluble phenolic compounds and lipid-soluble diterpenoid compounds, whose pharmacological properties have been studied for more than 60 years. Danshensu, salvianolic acid A (Sal A), and salvianolic acid B (Sal B) are phenolic caffeic acid derivatives, and idiographic water-soluble active components of *S. miltiorrhiza.* Danshensu [3-(3,4-dihydroxyphenyl)-2-hydroxy propanoic acid] is a relatively simple phenolic acid that exhibits antioxidant activity [5,6]; additionally, it exhibits higher scavenging activity against free radicals (for example, hydroxyl radicals (•OH), superoxide anion radicals (O2•−), and 1,1-diphenyl-2-picrylhydrazyl (DPPH) radicals) than vitamin C does [5].

Danshensu has major clinical applications in cardiovascular protection. Recently, the therapeutic and medicinal potential of danshensu as a hepatoprotective [7], neuroprotective [8], anti-corneal inflammatory [9], and anti-cataractogenic [10] agent has also been proposed. In particular, the use of danshensu in central nervous system disorders as anxiolytic medication and neuroprotective supplement is associated with either increase in dopamine (DA) level [11] or modulation of the dopamine D1 receptor [12]. Dopamine deficiency has also linked to pathological conditions of oculo-visual abnormalities, involving visual dysfunction, dynamic contrast sensitivity, color discrimination, and visual processing speeds that usually been described in patients with Parkinson’s disease (PD) [13,14,15], or diabetic retinopathy (DR) [15]. An animal study indicated that dopamine D1 or D4 receptor agonists could improve the functional vision of spatial frequency or contrast sensitivity in the early stage of diabetic retinopathy [16]. Chinese herbal formulae containing *S. miltiorrhiza* have been used as potential therapeutic agents to improve neuroretinal disease in ethnopharmacological clinical applications [17]. Borneol is a messenger drug used traditionally to facilitate the transport of pharmaceutical components to specific tissues, thus harmonizing the drug’s effects [18,19]. The intraocular delivery of danshensu was developed and improved using borneol to enhance blood-retinal barrier permeability [20]. However, the effect of danshensu in retinal protection and its functional regulation have not been fully studied.

In a mouse model, light-induced retinal photodamage and degeneration were observed, due to the rapid generation of reactive oxygen species. Several studies have shown that supplementation with nutrients or antioxidants could delay the onset of or attenuate photoreceptor injury [21,22,23]. Rhodopsin, M-opsin, and S-opsin are important functional proteins localized in the outer segment of the photoreceptor, that regulate photoreceptor response and primary vision processes. High energy light-evoked mislocalization of opsin protein causes progression of retinal dysfunction or functional vision abnormal changes [23]. In the current study, danshensu was tested in a mouse model of light-induced retinal damage to investigate its retinal protective effect and to explore its clinical applications in vision protection.

## 2. Materials and Methods

### 2.1. Animal

Female BLTW:CD1(ICR) mice (n = 70), 9–10 weeks old, were purchased from BioLASCO Taiwan Co., Ltd. (Taipei, Taiwan). The mice were housed in a temperature (23 ± 2 °C) and humidity-controlled room (55 ± 7%). The mice had free access to food and water. The experimental protocol was reviewed and approved by the Institutional Animal Care and Use Committee (IACUC1949) of Chung Shan Medical University based on the Guide for the Care and Use of Laboratory Animals.

### 2.2. Experimental Design and Animal Grouping

**Figure 1:**
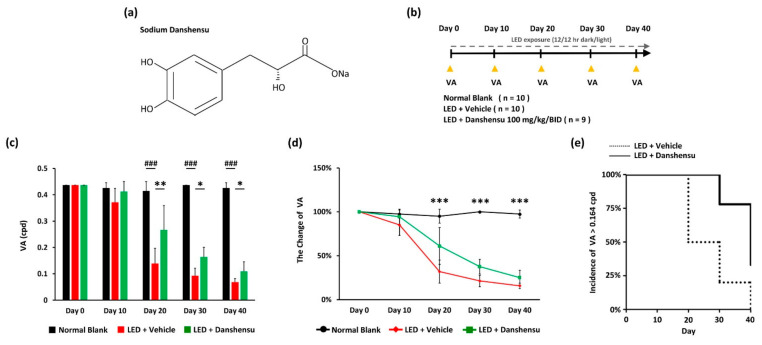
Effect of danshensu on light-evoked visual acuity (VA) deterioration. (**a**) Chemical structure of sodium danshensu; (**b**) Timeline of experimental design; (**c**) Representation of the VA threshold. Data are mean ± SE. Mann-Whitney U test. ^###^ *p* < 0.001, compared with the blank group. ** *p* < 0.01, and * *p* < 0.05, compared with the vehicle group; (**d**) Relative change in VA. Data are mean ± SE. Kruskal–Wallis test. *** *p* < 0.001; (**e**) Incidence rate of VA > 0.164 cpd.

Mice were randomly assigned to three groups: One blank group (n = 10) and two light exposure groups; (1) light-emitting diode (LED) + vehicle treatment group (n = 10) and (2) LED + danshensu treatment group (100 mg/kg, BID) (n = 9). 90% (*v*/*v*) distilled water containing 10% Propylene glycol 400 (PEG 400) is used as a vehicle for oral administration. The vehicle and danshensu groups were administered by oral gavage daily for 40 days. Sodium danshensu (Figure 1a) was purchased from ChemFaces (China). A light-induced retinal damage model was established as described in our previous study with minor modification of light stimulation performed for 40days (Figure 1b) using an LED light bulb (600–1000 lux, 12 h:12 h light-dark cycle) [23]. Mice were kept in dim conditions (50 ± 10 lux of illuminance) for 30 min before the functional visual examination, including the assessment of visual acuity (VA) and visual contrast sensitivity function (VCSF), or until they were sacrificed. In the pilot study (Supplemental Figure S1), the dosage of danshensu was tested and modified according to the animal study of Danshensu Bingpian Zhi [24]. The danshensu-treated mice (100 mg/kg, BID) had better VA performance on days 20 (Supplemental Figure S1). Therefore, the recommended dose of danshensu was given orally twice at 100 mg/kg in this study.

To explore whether danshensu participates in the dopaminergic pathway that contributes to functional vision. D1 receptor antagonist (SCH 23390) was purchased from Cayman Chemical Company (Ann Arbor, Michigan, USA) and dissolved in saline for injecting into mice. Light-evoked damage was generated in mice using the method described above, and mice were randomly assigned to three groups (Figure 5a): (1) LED + vehicle treatment group (n = 3), (2) LED + danshensu treatment (100 mg/kg, BID) + saline injection group (n = 6), and (3) LED + danshensu treatment (100 mg/kg, BID) + SCH 23390 (0.05 mg/kg) injection group (n = 6). The mice received saline or SCH 23390 injection on day 20 after danshensu treatment. Functional visual examination was performed before and after 30 min of saline or SCH 23390 injection. The procedures and criteria of SCH 23390 (0.05 mg/kg) injection was modified according to methods described in a mouse study [25]. The experimental dose of SCH 23390 (0.05 mg/kg) was selected owing to the fact that it does not interfere with the VA threshold under normal conditions, which was confirmed in blank mice (Supplemental Figure S2).

### 2.3. Determination of Thresholds of Visual Acuity (VA) and Visual Contrast Sensitivity Function (VCSF)

The methods for functional vision tests determining VA and VCSF were established as described in our previous study [23]. In the rapid evaluation, the thresholds of VA and VCSF in mice were determined based on reflexes that elicit compensatory head movements when the visual fields of the mice were exposed to rotating striped grating patterns (optomotor response, OMR) in unrestrained conditions [26,27,28,29,30]. The striped grating patterns were set at 100% contrast in the VA test. A square wave of the striped grating pattern was displayed. Then, stimulation was performed at different spatial frequencies of 0.033, 0.055, 0.082, 0.164, 0.328, and 0.437 cycles per degree (cpd) with a constant rotational speed (12°/s) drifting horizontally. The observer recorded OMR until they were no longer coordinated with the stimulus gratings, and hence, the VA threshold level of the mouse was determined. The VCSF test was performed according to the method described in our previous study [23] at the spatial frequencies described earlier; the striped grating patterns were set at ten different contrast levels to determine the threshold. The inverted U-shaped curve of VCSF was obtained using the described six-striped grating patterns of spatial frequencies with different thresholds of contrast levels and represents the capacities and characterization of spatial frequency-based vision. The area of the inverted U-shaped curve was converted to a VCSF visibility index, which represents the overall VCSF performance. However, if mice respond to relatively lower-contrast stimuli, additional capacities of functional vision could be calculated.

### 2.4. Histological Analyses and Immunohistochemistry

The mice were sacrificed in accordance with the regulations of the Guide for the Use of Laboratory Animals after the final examination of functional vision. Then, the eyeball was enucleated and fixed using fixative solution (3% formaldehyde, 5% glacial acetic acid, and 44% ethanol) before being embedded in paraffin. Paraffin sections of 5 μm thickness were prepared and stained with hematoxylin and eosin (H&E). The retinal microstructures were analyzed under an Olympus CX-22 microscope (Olympus Corp., Tokyo, Japan). The digitized image of the entire retina was scanned using a Motic Moticam 3 camera and Motic Image Plus v. 2.0 software (Motic, Xiamen, China) at 200× magnification. The nuclei of the outer nuclear layer (ONL) and the layer thickness of outer segments-inner segments (OS-IS) from each experimental group were measured and vertically plotted at 0.2, 0.4, 0.6, 0.8, 1.0, 1.2, 1.4, and 1.6 mm superior and inferior to the optic nerve head (ONH). For immunohistochemistry, anti-M opsin antibody (1/500, Cat. No. NB110-74730, Novus Biologicals, Littleton, CO, USA), anti-rhodopsin antibody (1/800, Cat. No. ab98887, Abcam, Cambridge, UK), anti-retinal pigment epithelium 65 (RPE65) antibody (1/600, Cat. No. NB100-355, Novus Biologicals, Littleton, CO, USA), and anti-glial fibrillary acidic protein (GFAP) antibody (1/400, Cat. No. ab7260, Abcam, Cambridge, UK) were incubated with retinal sections after antigen retrieval as per the citrate buffer (pH 6.0) method [23], and were then detected using an immunohistochemistry kit (Super Sensitive™ Polymer-HRP IHC Detection System, BioGenex Laboratories, Inc., Fremont, CA, USA). The average values of the cells labeled as M opsin, GFAP, or RPE65 were graphed in the superior and inferior retinas. M opsin mislocalization index was graphed as a percentage of the number of M opsin-labeled cells localized in the ONL profile according to their relative superior and inferior retinal positions.

### 2.5. Statistical Analysis

Quantitative data are expressed as mean ± standard deviation (SD). Statistical tests were performed using SPSS v. 22 software (IBM Corp., Armonk, NY, USA). Differences between groups were analyzed using the Kruskal–Wallis test and Mann–Whitney U-test. Association between the VA or VCSF and the residual ONL and OS-IS layers were assessed by the Pearson’s correlation test. A significant effect was defined as *p* < 0.05, *p* < 0.01, and *p* < 0.001.

## 3. Results

### 3.1. Effect of Danshensu on the Progression of Light-Evoked Visual Acuity Deterioration

Compensatory eye or head reflex (OMR) behavior is based on integrating information from the shifts in the retinal visual image. The examination of responses elicited through different spatial frequencies provided a VA threshold estimate in the mouse. The protective effect of danshensu on the progression of vision deterioration was observed in an early phase of the experimental light-induced retinal photodamage model (Figure 1c). During the early phase on day 20, danshensu-treated mice with retinal photodamage underwent a significantly higher VA threshold than the vehicle group (*p* < 0.01; Figure 1c). Moreover, the protective effect of danshensu against the progression of VA deterioration was observed during the late phase on days 30 and 40 (Figure 1d). There were significant differences between the danshensu and vehicle groups on days 30 (*p* < 0.05; Figure 1c) and 40 (*p* < 0.05; Figure 1c). The incidence rate of VA threshold higher than 0.164 cpd varied from the initial value and decreased in both LED photodamage groups throughout the experimental period after day 20 (Figure 1e). However, danshensu treatment resulted in a significant decrease in the rate of progression of vision deterioration compared to the vehicle group in the early condition of retinal photodamage (Figure 1c,d).

### 3.2. Protection of Low Spatial Frequency Vision against High Energy Light-Evoked Vision Damage by Danshensu

**Figure 2:**
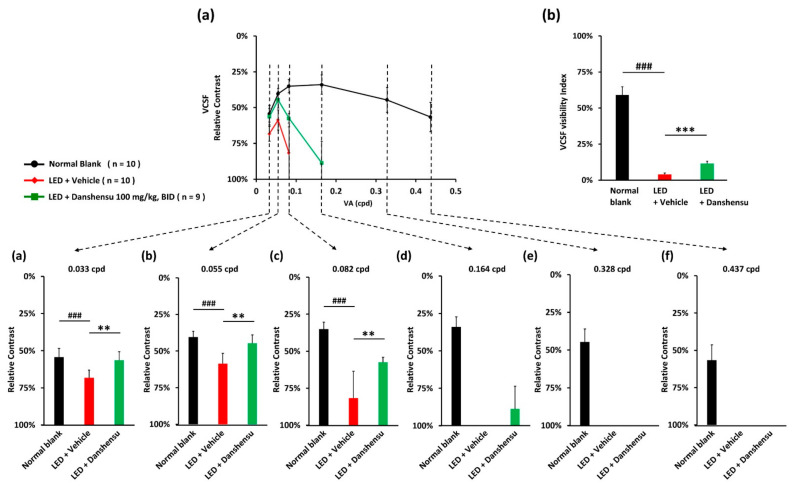
Protection of low spatial frequency-based visual contrast sensitivity function (VCSF) by danshensu treatment. (**a**) Representation of the inverted U-shape diagram of VCSF curve; (**b**) VCSF visibility index. Individual threshold with details represented in 0.033 cpd (**a**), 0.055 cpd (**b**), 0.082 cpd (**c**), 0.164 cpd (**d**), 0.328 cpd (**e**), and 0.437 cpd (**f**). Data are mean ± SD, Mann–Whitney U test, N.S., non-significant. ^###^ *p* < 0.001, compared with the blank group. *** *p* < 0.001, and ** *p* < 0.01, compared with the vehicle group.

The VCSF threshold was determined by a wave grating using various relative contrasts as a function of spatial frequency, whereas if mice respond to relatively lower-contrast stimuli, additional capacities of functional vision could be calculated. The curve of the VCSF threshold usually forms an inverted U-shape with a peak (Figure 2a). The VCSF visibility index was calculated using the area under the inverted U-shaped curve, which increased with an increase in VCSF performance (Figure 2b). To distinguish the efficacy on different spatial frequency-based vision, the VCSF thresholds were determined individually as 0.033 cpd (Figure 2a), 0.055 cpd (Figure 2a,b), 0.082 cpd (Figure 2a–c), 0.164 cpd (Figure 2a–d), 0.328 cpd (Figure 2a–e), and 0.437 cpd (Figure 2a–f). On day 40, the danshensu group showed better VCSF curves than the vehicle group (Figure 2a). However, danshensu treatment resulted in the loss of high spatial frequency-based VCSF (Figure 2a–f). In contrast, low spatial frequency-based VCSF was particularly protected, indicating that danshensu significantly improved the thresholds especially at low spatial frequencies like 0.033 cpd at 56.41 ± 5.69% (Figure 2a), 0.055 cpd at 44.65 ± 5.89% (Figure 2a,b), and 0.082 cpd at 57.47 ± 3.32% (Figure 2a–c), respectively, relative to the LED-vehicle group at 68.26 ± 5.19% (Figure 2a), 58.65 ± 7.11% (Figure 2a,b), and 81.73 ± 18.26% (Figure 2a–c) (*p* < 0.01). Particularly, at the middle-level spatial frequency of 0.164 cpd, the VCSF threshold of the danshensu group was 88.67 ± 15.09%, while it was not detectable in the vehicle group (Figure 2a–d). The VCSF visibility index was substantially lower in the vehicle group (3.98 ± 0.92%), but slightly increased in the danshensu group (11.42 ± 1.73%; *p* < 0.001; Figure 2b). These results indicated that danshensu primarily protected low spatial frequency-based visual performance in a mouse model of the photo-stressed retina.

### 3.3. Prevention of Müller Cell Gliosis by Danshensu

**Figure 3:**
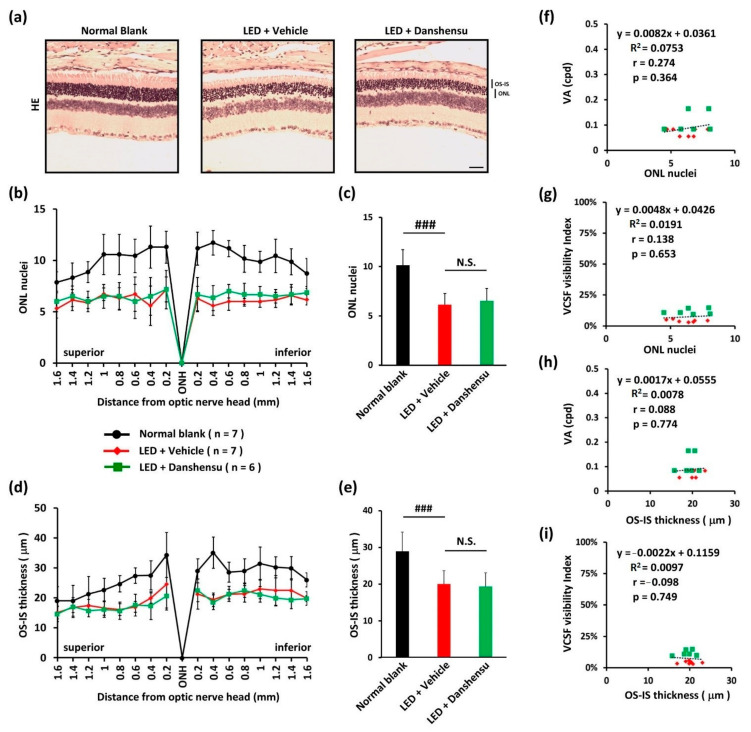
Effect of danshensu on light-evoked retinal damage. Representation of H&E staining (**a**). The residual thickness of ONL (**b**) and OS-IS (**d**) were measured within 1.6 mm superior and inferior to the optic nerve. The average thickness of ONL (**c**) and OS-IS (**e**) were analyzed and quantified within 1.0 mm superior and inferior to the optic nerve. The correlation analysis between VA and ONL thickness (**f**), VA and OS-IS thickness (**g**), VCSF visibility index and ONL thickness (**h**), and VCSF visibility index and OS-IS thickness (**i**). Data are expressed as mean ± SD. Mann–Whitney U test (C and E), N.S., non-significant. ^###^ *p* < 0.001, compared with the blank group. Scale bar: 35 μm.

We assessed the loss of photoreceptors through histopathologic microscopic examination of the whole retina on day 40 (Figure 3a). The loss of photoreceptor nuclei in ONL (Figure 3b,c) and OS-IS did not differ significantly in the danshensu group from that in the retinas of the vehicle group (Figure 3d,e), indicating no effect of danshensu on light-evoked tissue damage to retinas. Moreover, the morphology of the residual ONL or OS-IS was not correlated with the residual threshold of VA or VCSF (Figure 3f–i).

**Figure 4:**
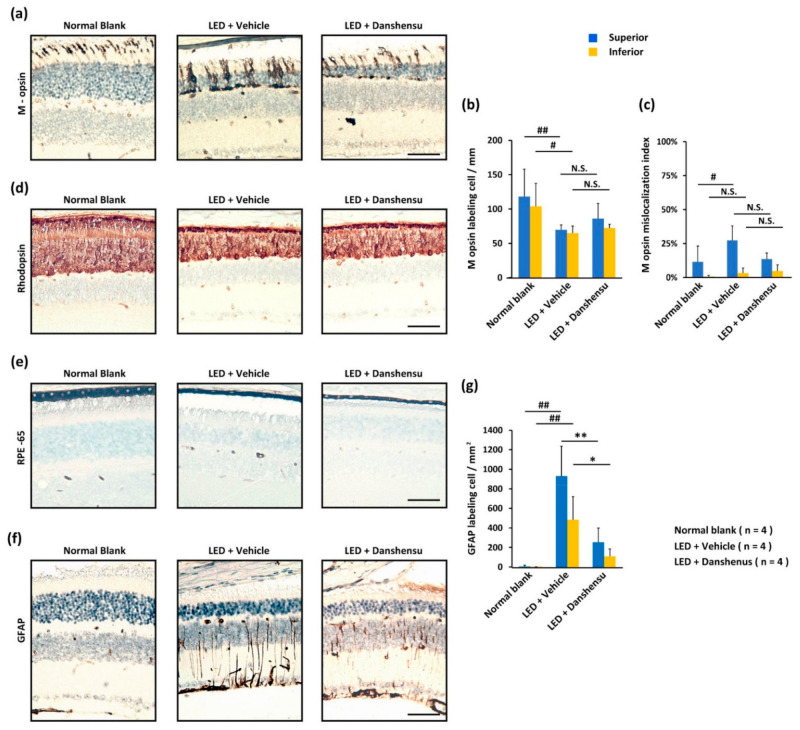
Effect of danshensu on cellular protection after light-evoked retinal damage. (**a**) IHC staining showing the alterations of the photoreceptor-specific function of M opsin protein; (**d**) and Rhodopsin in the retinas; (**b**) Representation of M opsin-labeled cell density; (**c**) and the percentage of M opsin mislocalization; (**e**) Alterations in the pigment cell layer of outer blood-retinal-barrier (outer BRB) when labeled with RPE65 protein; (**f**) The pathologic Müller cells of inner blood-retinal-barrier (inner BRB) when labeled with GFAP protein; (**g**) Representation of the density of GFAP-labeled cells. Data are mean ± SD. Mann–Whitney U test, N.S., non-significant. ^##^ *p* < 0.01, ^#^ *p* < 0.05, compared with the blank group. ** *p* < 0.01, and * *p* < 0.05, compared with the vehicle group. Scale bar: 35 μm.

When stained with cone-specific antibodies (Figure 4a), the number of M opsin-labeled cones (Figure 4b) was similar in the retinas of the danshensu and vehicle groups. Light-evoked M opsin mislocalization, which dominates labeling in ONL, was observed in the superior retinas of the vehicle group compared to that in the danshensu group (Figure 4c). However, the difference was not significant (Figure 4c). The labeling expression of rod-specific antibodies, rhodopsin, was similar in the residual ONL or residual OS-IS layer of retinas in danshensu and vehicle groups (Figure 4d). These experiments demonstrated that photoreceptors did not serve as targets of the cellular protective effect exerted by danshensu.

In particular, a thinner change in the RPE65-labeled pigmented layer was observed in the retinas of the danshensu and vehicle group compared to the blank group (Figure 4e). Light-evoked Müller cell gliosis was detected by GFAP staining, whereas characteristic changes subsequently leading to dystrophies in retinas have been well documented. Danshensu prevented Müller cell gliosis (Figure 4f), which significantly reduced GFAP labeling in cells compared to the vehicle group (Figure 4g) with significant differences in both superior retinas (*p* < 0.01) and inferior retinas (*p* < 0.05) (Figure 4g). These results indicated that the pigmented layer and Müller cells might alternatively serve as protective targets of danshensu compared to photoreceptors in retinas.

### 3.4. Improvement of VCSF by Danshensu via Modulation of Dopamine Receptor

**Figure 5:**
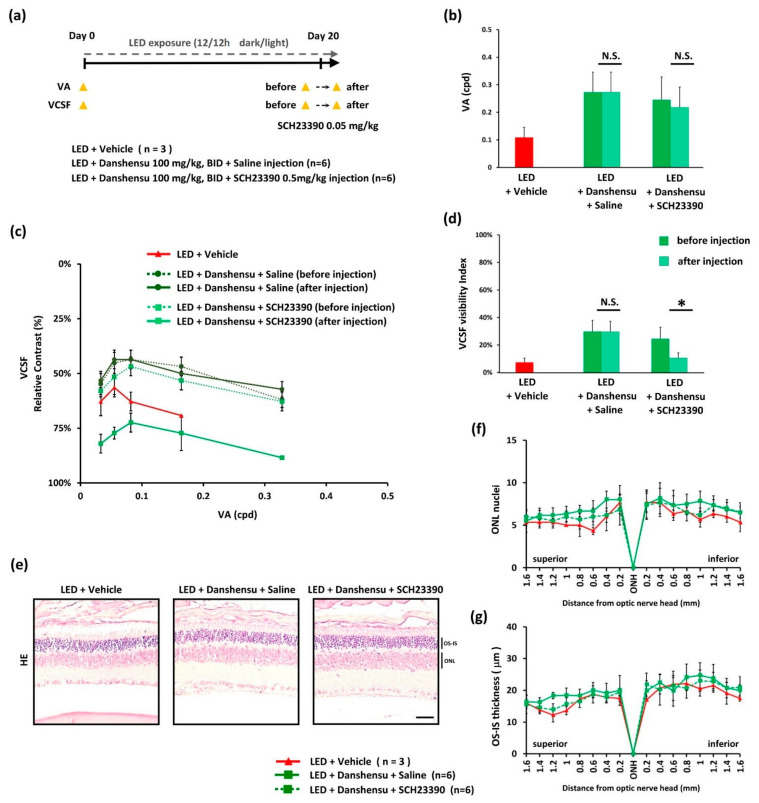
Effect of danshensu on the modulation of dopamine receptor to improve VCSF. (**a**) Timeline of experimental design; (**b**) Representation of the change in VA threshold; (**c**) Change in VCSF threshold; (**d**) Change in VCSF visibility index after dopamine D1 receptor antagonist (SCH 23390) injection; (**e**) Representation of the absence of difference after H&E staining; (**f**) Residual thickness of ONL (**g**) and OS-IS after SCH 23390 injection. Data are expressed as mean ± SE. Mann–Whitney U test, N.S., non-significant. * *p* < 0.05, compared with the vehicle group. Scale bar: 35 μm.

Danshensu functionally elevates the threshold of low spatial frequency vision according to the above findings. Although the vision protective effect of danshensu was demonstrated to be independent of retinal tissue integrity, it might functionally elevate the performance of the residual photoreceptor and/or the secondary vision-bases neural processing system. On day 20, the threshold of VA and VCSF, as described above, was lower in the vehicle-treated group than in the danshensu-treated group (Figure 5b,d). After injection with a dopamine D1 receptor antagonist (SCH 23390) in the danshensu-treated group, the protective level of danshensu on the VCSF threshold was significantly reversed (*p* < 0.05) (Figure 5c). Moreover, the VCSF visibility index declined significantly after injection with dopamine D1 receptor antagonist (SCH 23390) (Figure 5d). However, similar results were not observed in the danshensu-treated group after saline injection (Figure 5c,d). Under these conditions, the threshold of VA was not significantly changed (Figure 5b), and without obvious histological changes in retinas (Figure 5e–g). These results indicate that the vision protective properties of danshensu might be exerted via the modulation of the dopamine D1 receptor.

## 4. Discussion

High energy light-evoked retinal oxidative damage has been recognized as a contributing factor to the pathogenesis of clinical retinopathy [21]. To explore the protective role of danshensu against light-evoked visual damage in vivo, a mouse model of light-induced retinal photodamage was used. We studied the protective efficiency and limitation of danshensu on functional vision and retinal structure integrity and described the potential mechanisms associated with its vision promotion effect.

Danshensu is an active component of the aqueous extract of *S. miltiorrhiza*, a traditional Chinese herb, which has potential applications in the food and healthcare industry to promote cardiovascular health promotion [5]. Clinical studies have reported that danshensu exhibits various bioactivities and therapeutic uses, including applications in cardiovascular diseases (for example, myocardial ischemia, atherosclerosis, and hypertension), cerebral disorders (for example, ischemia, cognitive disorders, and anxiety), and tumors-treating fields [29]. In vivo and in vitro studies have revealed the underlying mechanisms of the protective effects or therapeutic uses of danshensu, including the suppression of reactive oxygen species generation [6], as radical scavengers and regulation of antioxidant response [5], inflammation regulation [29,30], vessel tension regulation [31], hyperlipidemia control [29], dopamine release, and dopaminergic signaling promotion [11,12].

Traditionally, such formulae of Chinese herbs were known to lower the risk of developing microvascular diabetic complications in retinas and to prevent their progression [17]; these effects have been confirmed in animal studies [32,33,34]. Additionally, enhancing the bioavailability of danshensu in intraocular fluid (aqueous humor and vitreous humor) using borneol delivery has been reported [20]. In addition, a study has indicated that the anti-oxidative property of danshensu might help prevent cataractogenesis [10]. Recently, we also reported the anti-inflammatory property of danshensu in the cornea against UVB damage via oral administration [9]. However, there is no research to date, which has discussed the effects of danshensu on retinas, photoreceptors, and vision performance.

The present study demonstrated that danshensu cannot directly contribute to photoreceptor protection against light-evoked tissue damage, but can significantly protect the Müller cells from injury. Retinal pigmented cells and retinal Müller cells, which support the interactions between retinal neurons for homeostasis, nutrition, and metabolism, play key roles in the blood-retinal-barrier (BRB) function. In particular, Müller gliosis indicated by GFAP staining reflects the severity of the oxidative damage of retinal neurons [35,36], which leads to photoreceptor degeneration, intraretinal vascular leak, and microvasculopathy. Additionally, the mislocalization of M opsins is the consequence of a defect in the chromophore 11-cis retinal provided by the retinal pigmented cells and retinal Müller cells involved in visual cycles. Nevertheless, our study found that danshensu has no clear effect on retinal pigmented cells. According to the results found in the present study, danshensu does not significantly decrease opsins mislocalization, and it is conjectured that these events might contribute to loss of the vision protective efficacy of danshensu in the late phase of this experimental model. Light-evoked oxidative free radicals can also induce photoreceptor apoptosis, but danshensu cannot stop cell loss, including cones and rods. Nonetheless, danshensu treatment attenuated the progression of light-evoked deterioration of VA in the early phase; however, it eventually developed a relatively lower threshold level of VA, which indicates that the degeneration of ONL and OS-IS has not strictly been restricted and regulated by danshensu. On the other hand, the VA protective effect by dopamine D1 receptor-dependent pathway was not been compensated, due to more damage of retinal pigmented cells in the late phase of this model. Based on these findings, it is suggested that danshensu might partly contribute to photoreceptor function by an unknown molecular mechanism, but might not be cellular structurally maintained. However, this will have to be proved through further research.

It is well known that functional vision improves by nutrients or active components, which are useful for enhancing primary visual processes, secondary visual processes, and provide additional neurotransmitters associated with vision. Recently, studies have reported the contribution of dopamine in optimizing visual processing under photopic or scotopic conditions [37,38], especially through the rods [39], and the sensitivity of the spatial selectivity of visual processing [40,41]. Furthermore, dopamine receptors can modulate visual processing [40,41] or VCSF and VA performance [42] in mice. Danshensu treatment could increase the dopamine level in the brain [11], and contribute to an anxiolytic-like effect in mice via activation of the dopamine D1 receptor [12]. Subsequently, we determined whether danshensu increases the capacity of residual photoreceptor function to enhance VCSF by modulating dopamine receptors. We observed that dopamine D1 receptor antagonist (SCH 23390) injection can significantly reverse the VCSF level, which was improved by danshensu as compared to saline injection in the danshensu-treated group, during the early phase of light-evoked retinal damage on day 20. Experimentally, when therapeutic treatment by danshensu was started on day 30 during the late-stage light-induced retinal injury, it did not provide significant restorative effects on residual VA and VCSF with less structural integrity (Supplemental Figure S3). Overall, these results confirmed and indicated that the vision protective property of danshensu might occur via modulation or activation of the dopamine D1 receptor based on visual processing with enough associated healthy cells, although it did not dominantly protect the retinal structure integrity.

In summary, our in vivo results demonstrated the effects of danshensu on retinas and functional vision in a mouse model of light-induced retinal degeneration. The protective effect of VCSF is the result of its enhancement associated with the dopamine D1 receptor. In conclusion, danshensu is still a potential candidate for eye healthcare, which may participate in the protection of the blood-retinal-barrier (BRB) or vision; the effects are mainly associated with dopaminergic enhancement, rather than the protection of retinal tissue structure.

## Data Availability

Data available only on request due to ethical restrictions.

## Supplementary Materials

The following are available online at https://www.mdpi.com/2072-6643/13/3/978/s1, Figure S1: Oral administration of danshensu retarded the decline of visual acuity (VA) in a mouse model of light-induced retinal damage. Figure S2: Determination of the threshold of visual acuity (VA). Figure S3: Effect of oral administration of danshensu (100 mg/kg, BID) in mice on day 30 after light-induced retinal damage.
