# Supplementary material for: The Functional Vision Protection Effect of Danshensu via Dopamine D1 Receptors: In Vivo Study
Source: Nutrients. 2021 Mar 17;13(3):978. doi: 10.3390/nu13030978 (PMC8002943; doi:10.3390/nu13030978)
Supplement: Supplementary file 1 [file nutrients-13-00978-s001.pdf]

nutrients

MDPI

Article

The Functional Vision Protection Effect of Danshensu via Dopamine D1 Receptors: In Vivo Study

Yun-Wen Chen [1], Yun-Ping Huang [2], Pei-Chang Wu [1], Wei-Yu Chiang [1], Ping-Hsun Wang [2] and Bo-Yie Chen [2,3,*]

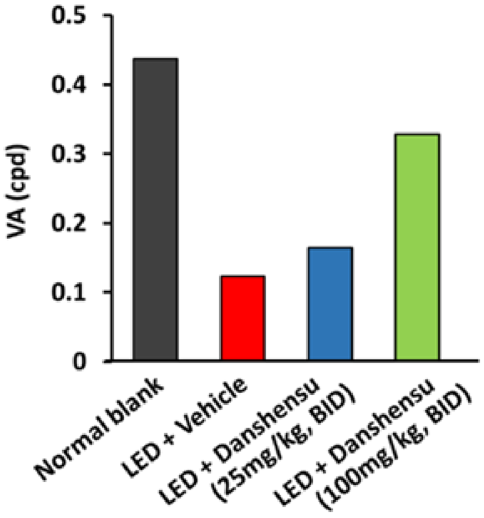

Figure S1. In the pilot study, oral administration of danshensu retarded the decline of visual acuity (VA) on the day20 in a mouse model of light-induced retinal damage. The vehicle-treated mouse (n=2) showed poorer visual acuity compared to the normal blank group (n=2). The visual acuity threshold in danshensu (100mg/kg, BID)-treated mice (n=2) are better than danshensu (25mg/kg, BID) -treated mice (n=2). Results are presented as means ± SEMs.

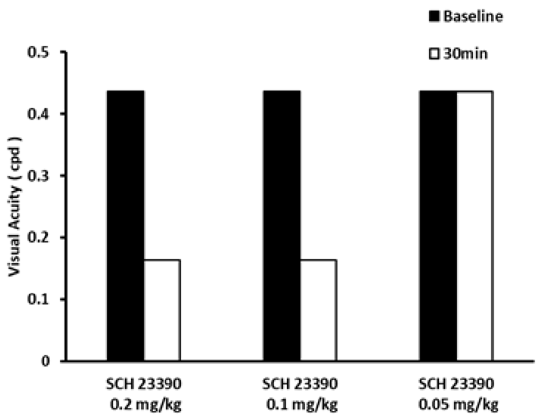

Figure S2. Determination of the threshold of visual acuity (VA). In the blank mice group, injection of SCH 23390 (dopamine D1 receptor antagonist) at 0.05 mg/kg did not interfere with the threshold of visual acuity (VA). VA determination was performed before SCH 23390 injection and 30 min after the injection.

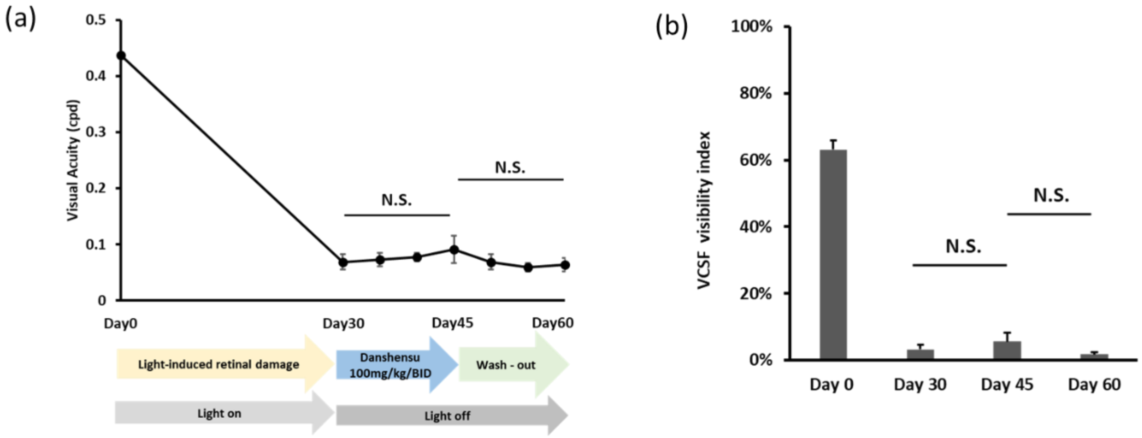

Figure S3. Effect of oral administration of danshensu (100 mg/kg, BID) in mice (n = 6) on day 30 after light-induced retinal damage. The damaged retinas were characterized by the additional loss of structural integrity on day 30. The residual VA threshold (a) and VCSF visibility index (b) were not rescued by danshensu under these conditions. Data are expressed as mean ± SD. Mann–Whitney U test, N.S., non-significant.
